# Prognostic Value of the Modified Endothelial Activation and Stress Index (m-EASIX) for 90-Day Mortality in Patients with Moderate-to-Severe Hypercalcemia of Malignancy

**DOI:** 10.3390/jcm15197382

**Published:** 2026-09-23

**Authors:** Nevriye Gül Ada Tak, Hikmet Öztop, Fazıl Çağrı Hunutlu

**Affiliations:** 1 Department of Internal Medicine, Bursa Buyukorhan Integrated State Hospital, Bursa 16990, Turkey; 2 Department of Internal Medicine, Faculty of Medicine, Bursa Uludag University, Bursa 16059, Turkey; hikmetoztop@uludag.edu.tr; 3 Department of Hematology, Bursa City Training and Research Hospital, University of Health Sciences, Bursa 16280, Turkey; fazilhunutlu@gmail.com

**Keywords:** hypercalcemia of malignancy, m-EASIX, endothelial activation, prognosis, mortality, biomarker

## Abstract

**Background:** Hypercalcemia of malignancy (HCM) is associated with poor short-term prognosis, particularly when it represents the initial manifestation of an underlying, as yet uncharacterized malignancy. Serum calcium concentration alone may not adequately capture the systemic biological burden associated with advanced disease. **Methods:** In this retrospective, single-center cohort study, 71 patients with moderate-to-severe hypercalcemia (corrected calcium ≥12 mg/dL) in whom a hematologic or solid malignancy was subsequently diagnosed were evaluated. The modified Endothelial Activation and Stress Index (m-EASIX = LDH × CRP/platelet count) was calculated at presentation. Univariate and multivariable logistic regression, receiver operating characteristic (ROC) analysis and Kaplan–Meier survival analysis were used to evaluate the association between Log2 m-EASIX and 90-day mortality. **Results:** Log2 m-EASIX remained independently associated with 90-day mortality in multivariable analysis (adjusted OR = 1.96, 95% CI 1.37–2.82, *p* < 0.001), whereas white blood cell count and albumin did not reach statistical significance in the same model. Corrected serum calcium was not associated with mortality (*p* = 0.702). Log2 m-EASIX showed good discriminative performance (AUC = 0.857, 95% CI 0.768–0.946) and stratified 90-day survival in both hematologic (*p* < 0.001) and solid malignancies (*p* = 0.002), with no significant interaction between m-EASIX and malignancy subgroup (*p* = 0.499). **Conclusions:** m-EASIX, calculated from routinely available laboratory parameters, may provide clinically useful prognostic information for early risk stratification in patients presenting with hypercalcemia of malignancy.

## 1. Introduction

Hypercalcemia of malignancy (HCM) represents a clinically significant metabolic complication of cancer, particularly in advanced disease, and is frequently associated with unfavorable clinical outcomes [1]. The underlying mechanisms of HCM are diverse, including humoral effects mediated by parathyroid hormone-related peptide, calcium release linked to local osteolysis, excessive production of 1,25-dihydroxyvitamin D and, less commonly, ectopic secretion of parathyroid hormone [2,3]. Moderate or severe hypercalcemia can affect multiple organ systems, resulting in manifestations such as volume depletion, renal dysfunction and gastrointestinal, neurological and cardiovascular abnormalities [4,5,6]. Nevertheless, short-term mortality continues to remain substantial among patients presenting with HCM [7,8].

Although serum calcium concentration defines the biochemical severity of hypercalcemia, prognosis in HCM is likely determined by multiple factors related to the underlying malignancy and the systemic response to disease [9]. Inflammation, cellular injury and turnover, and alterations in endothelial and coagulation pathways may reflect aspects of disease burden that are not captured by serum calcium alone [10]. Readily available laboratory-based indices integrating these processes may therefore provide additional information for early risk stratification. Such an approach may be particularly relevant when hypercalcemia is the presenting manifestation of an otherwise unrecognized malignancy, because tumor type, disease extent and malignancy-specific prognostic characteristics may not yet be known at the time of initial assessment.

The Endothelial Activation and Stress Index (EASIX) was initially introduced to quantify biological processes related to endothelial dysfunction and systemic stress using routinely measured laboratory variables; its potential prognostic role has since been explored in several clinical contexts [11]. Modified versions of EASIX have been proposed to incorporate systemic inflammatory activity. The modified EASIX (m-EASIX), which combines C-reactive protein (CRP), lactate dehydrogenase (LDH) and platelet count, integrates laboratory parameters related to systemic inflammation, cellular injury and turnover, and endothelial-related processes. The prognostic value of EASIX- and m-EASIX-based indices has been demonstrated in several hematologic and solid malignancies [12,13,14,15]. However, their prognostic utility in patients presenting with HCM has not been well characterized.

Available HCM studies have predominantly examined hypercalcemia arising after a cancer diagnosis has already been established. Comparatively limited information is available regarding prognostic markers in patients in whom moderate-to-severe hypercalcemia represents the initial clinical manifestation leading to the diagnosis of an underlying malignancy [16]. In this setting, a simple index derived from routinely available laboratory parameters could potentially facilitate early risk assessment while the diagnostic work-up and characterization of the underlying malignancy are still ongoing.

Therefore, the present study aimed to evaluate the association between m-EASIX and 90-day mortality in patients presenting with moderate-to-severe hypercalcemia in whom an underlying hematologic or solid malignancy was subsequently diagnosed. We also assessed the discriminatory performance of m-EASIX for 90-day mortality and explored whether its prognostic association differed between hematologic and solid malignancies.

## 2. Materials and Methods

### 2.1. Study Design and Patients

This retrospective, single-center cohort study included patients who presented with hypercalcemia between January 2020 and December 2025 at the Department of Internal Medicine, Bursa Uludag University. Patients were identified by screening the hospital’s electronic medical records system for a corrected serum calcium concentration above the upper limit of normal. Corrected calcium was calculated using the standard formula: corrected calcium (mg/dL) = measured total calcium (mg/dL) + 0.8 × [4.0 − serum albumin (g/dL)]. Patients with mild hypercalcemia (corrected calcium <12 mg/dL) were excluded, restricting the study population to those with moderate-to-severe hypercalcemia (corrected calcium ≥12 mg/dL) [1].

Among patients with moderate-to-severe hypercalcemia, those with primary hyperparathyroidism or hypercalcemia attributable to other non-malignant causes (e.g., drug-induced, granulomatous disease) were further excluded. The final study cohort comprised patients in whom hematologic or solid malignancy was diagnosed following presentation with hypercalcemia. The patient selection process is illustrated in Figure 1. Patients received standard supportive management for hypercalcemia according to clinical indication.

The research received approval from the Bursa Uludag University Clinical Research Ethics Committee (approval no. 2026/381/13-28) and was carried out in accordance with the Declaration of Helsinki. Because of the retrospective design, informed consent was not required.

### 2.2. Data Collection

Demographic characteristics, comorbidities, malignancy subtype and laboratory parameters obtained at presentation were retrospectively collected from hospital electronic medical records. Laboratory parameters included corrected serum calcium, parathyroid hormone, 25-OH vitamin D, albumin, total protein, phosphorus, white blood cell count, absolute neutrophil count, absolute lymphocyte count, absolute monocyte count, hemoglobin, hematocrit, platelet count, blood urea nitrogen, creatinine, lactate dehydrogenase (LDH), ferritin and C-reactive protein (CRP). Patients were categorized into two malignancy subgroups (hematologic vs. solid malignancy) based on the histopathological diagnosis established during the diagnostic work-up following presentation with hypercalcemia. Cause of death was retrospectively classified for patients who died within 90 days, based on discharge summaries and final clinical notes, as disease-related (disease progression, infection/sepsis, treatment-related toxicity or hypercalcemia-associated complications) versus non-disease-related or indeterminate (unrelated cardiac events, other unrelated causes or insufficient documentation). No missing data were present for any of the variables included in the analysis.

### 2.3. Definition of m-EASIX

The modified Endothelial Activation and Stress Index (m-EASIX) was calculated using the formula: m-EASIX = LDH (U/L) × CRP (mg/L)/platelet count (×10^9^/L) [17], using laboratory parameters obtained at presentation, prior to the initiation of hydration, bisphosphonate therapy or other hypercalcemia-directed treatment. Because raw m-EASIX values were markedly right-skewed, the base-2 logarithm of m-EASIX (Log2 m-EASIX) was used in all statistical analyses to satisfy the linearity assumption of logistic regression and to reduce the influence of extreme values.

### 2.4. Outcome

The primary outcome was all-cause mortality within 90 days of presentation with hypercalcemia (90-day mortality).

### 2.5. Statistical Analysis

Statistical analyses were performed using SPSS version 29.0 (IBM Corp., Armonk, NY, USA) and Python (version 3.12; Python Software Foundation; Wilmington, DE, USA; pandas, NumPy, SciPy, statsmodels, scikit-learn and lifelines libraries). Logistic regression and descriptive statistical comparisons were cross-validated between both platforms. Kaplan–Meier survival analysis, log-rank testing and the formal interaction analysis were performed in Python.

The normality of continuous variables was assessed using the Shapiro–Wilk test. Normally distributed continuous variables are presented as mean ± standard deviation and were compared using the independent samples t-test; non-normally distributed variables are presented as median (interquartile range) and were compared using the Mann–Whitney U test. Categorical variables are presented as frequencies and percentages and were compared using the chi-square test or Fisher’s exact test, as appropriate.

Univariate logistic regression analyses were performed using the enter method to assess the association between each candidate variable and 90-day mortality. Variables with *p* < 0.05 in univariate analysis were considered candidates for multivariable analysis. To avoid multicollinearity, neutrophil count was excluded because of its high correlation with white blood cell count, and CRP was excluded because it is an algebraic component of the m-EASIX formula. The remaining candidate variables were entered into a multivariable logistic regression model using the backward likelihood-ratio method. Odds ratios (ORs) with 95% confidence intervals (CIs) are reported for both univariate and multivariable analyses. A two-tailed *p*-value < 0.05 was considered statistically significant. Model performance was assessed using Nagelkerke R^2^ and the Hosmer–Lemeshow goodness-of-fit test.

Receiver operating characteristic (ROC) curve analysis was performed to evaluate the discriminative performance of Log2 m-EASIX for 90-day mortality, and the area under the curve (AUC) with 95% CI was calculated. The discriminatory performance of Log2 m-EASIX was also compared with that of CRP and NLR using pairwise DeLong tests for correlated ROC curves. The optimal cutoff value was determined using the Youden index, and the corresponding sensitivity and specificity were reported. Based on this cutoff, patients were categorized into m-EASIX-low and m-EASIX-high groups for survival analysis.

Kaplan–Meier survival analysis was used to estimate 90-day survival probabilities, with follow-up time censored at 90 days. Survival curves were compared using the log-rank test, both for the overall cohort stratified by malignancy subgroup and for the m-EASIX-low versus m-EASIX-high groups within each malignancy subgroup. To evaluate whether the prognostic effect of Log2 m-EASIX differed between hematologic and solid malignancies, a formal interaction analysis was performed by including a Log2 m-EASIX × malignancy subgroup interaction term in a logistic regression model, with statistical significance assessed using the likelihood ratio test.

A two-tailed *p*-value < 0.05 was considered statistically significant for all analyses.

## 3. Results

A total of 71 patients with moderate-to-severe hypercalcemia in whom an underlying malignancy was subsequently diagnosed were included in the final analysis, following the exclusion of patients with mild hypercalcemia (corrected calcium < 12 mg/dL, *n* = 92), primary hyperparathyroidism (*n* = 44) and other non-malignant causes of hypercalcemia (*n* = 32) from an initial screening population of 239 patients (Figure 1). The baseline demographic, clinical and laboratory characteristics of the study cohort are summarized in Table 1. The mean age was 60.8 ± 14.6 years and 40 patients (56.3%) were female. Thirty-one patients (43.7%) had a hematologic malignancy and 40 (56.3%) had a solid malignancy (Table 2). Multiple myeloma (*n* = 21, 29.6%) was the most common hematologic malignancy, whereas lung cancer (*n* = 16, 22.5%) was the most common solid malignancy. Cause of death, classified for the 36 patients who died within 90 days, was disease-related in 31 patients (86.1%; infection/sepsis, *n* = 19; disease progression, *n* = 11; treatment-related toxicity, *n* = 1), whereas death was non-disease-related or indeterminate in 5 patients (13.9%; Table 3).

### 3.1. Comparison Between Hematologic and Solid Malignancies

Patients with solid malignancies had significantly higher C-reactive protein, Log2 m-EASIX, hemoglobin, and neutrophil levels and significantly lower creatinine values, than patients with hematologic malignancies (Table 4). Total protein levels were significantly higher in the hematologic malignancy group. Ninety-day mortality was significantly higher among patients with solid malignancies (26/40, 65.0%) than among those with hematologic malignancies (10/31, 32.3%; *p* = 0.013).

### 3.2. Predictors of 90-Day Mortality

In univariate logistic regression analysis, age, neutrophil count, C-reactive protein, malignancy subgroup (hematologic vs. solid), total protein, albumin, white blood cell count and Log2 m-EASIX were significantly associated with 90-day mortality (all *p* < 0.05; Table 5). In contrast, corrected serum calcium concentration was not significantly associated with 90-day mortality (OR = 1.06, 95% CI 0.80–1.38, *p* = 0.702).

Neutrophil count was excluded from multivariable analysis because of its high correlation with white blood cell count, whereas C-reactive protein was excluded because it is an algebraic component of the m-EASIX formula. The remaining candidate variables were entered into a multivariable logistic regression model using the backward likelihood-ratio method. In the final model, Log2 m-EASIX remained independently associated with 90-day mortality (adjusted OR = 1.96, 95% CI 1.37–2.82, *p* < 0.001) corresponding to an approximate doubling of the raw m-EASIX value, whereas white blood cell count (OR = 1.13, 95% CI 0.99–1.30, *p* = 0.080) and albumin (OR = 0.89, 95% CI 0.78–1.01, *p* = 0.068) did not reach statistical significance. The final model showed a Nagelkerke R^2^ of 0.468 and good calibration according to the Hosmer–Lemeshow goodness-of-fit test (*p* = 0.795).

As sensitivity analyses, NLR was evaluated as an additional candidate variable but was eliminated during backward selection (*p* = 0.207), leaving the final model unchanged. Bootstrap resampling (1000 iterations) showed that Log2 m-EASIX was retained in 98.1% of resamples and Firth’s penalized logistic regression yielded a similar effect estimate (OR = 1.82, 95% CI 1.31–2.54, *p* < 0.001; Supplementary Tables S1 and S2). In subgroup analyses, Log2 m-EASIX remained significantly associated with 90-day mortality in both hematologic and solid malignancies (*p* = 0.012 and *p* = 0.004, respectively), whereas CRP and NLR lost significance in at least one subgroup (Supplementary Table S3).

### 3.3. Discriminative Performance and Cutoff Value

Receiver operating characteristic (ROC) curve analysis demonstrated good discriminatory ability of Log2 m-EASIX for 90-day mortality (AUC = 0.857, 95% CI 0.768–0.946, *p* < 0.001; Figure 2). Based on the Youden index, the optimal cutoff was identified as Log2 m-EASIX ≥6.17, corresponding to a raw m-EASIX value of 72.0, with a sensitivity of 83.3% (95% CI: 68.1–92.1%) and a specificity of 77.1% (95% CI: 61.0–87.9%). For comparison, the discriminative performance of CRP alone (AUC = 0.794, 95% CI 0.684–0.904) and NLR alone (AUC = 0.752, 95% CI 0.638–0.867) was also assessed. Pairwise comparisons using DeLong’s test showed that the differences in AUC between Log2 m-EASIX and CRP and between Log2 m-EASIX and NLR did not reach statistical significance (*p* = 0.123 and *p* = 0.138, respectively; Supplementary Table S4).

### 3.4. Survival Analysis

Kaplan–Meier analysis demonstrated significantly lower 90-day survival among patients with solid malignancies than among those with hematologic malignancies (35.0% vs. 67.7%; log-rank *p* = 0.002; Figure 3A). When patients were stratified according to Log2 m-EASIX using the cutoff of 6.17, significantly lower 90-day survival was observed in the m-EASIX-high group within both the hematologic malignancy subgroup (30.0% vs. 85.7%; log-rank *p* < 0.001; Figure 3B) and the solid malignancy subgroup (18.5% vs. 69.2%; log-rank *p* = 0.002; Figure 3C).

Consistent with these findings, univariate logistic regression showed that Log2 m-EASIX was significantly associated with 90-day mortality within each malignancy subgroup (hematologic: OR = 1.84, 95% CI 1.14–2.95, *p* = 0.012; solid: OR = 2.38, 95% CI 1.31–4.31, *p* = 0.004). A formal interaction analysis showed no significant interaction between Log2 m-EASIX and malignancy subgroup (*p* = 0.499), providing no evidence that the association between Log2 m-EASIX and 90-day mortality differed between hematologic and solid malignancies.

## 4. Discussion

The principal finding of this study was that the log2 m-EASIX score was independently associated with 90-day mortality in patients whose malignancy was diagnosed following presentation with hypercalcemia. Although several clinical, inflammatory and biochemical variables were associated with mortality in univariate analyses, m-EASIX retained its prognostic significance in multivariable analysis. In addition, m-EASIX showed good discriminatory ability for mortality, and higher m-EASIX levels were associated with poorer survival. The observation of this survival separation in both hematologic and solid malignancies suggests that the prognostic relevance of m-EASIX may extend across different malignancy categories.

Hypercalcemia of malignancy (HCM) is an important metabolic complication of advanced cancer and is closely associated with poor prognosis [7,18]. Previous studies have demonstrated high early mortality and limited survival among patients with HCM [8,19,20]. However, much of the existing literature is based on patients with an established diagnosis of malignancy who subsequently develop HCM during the course of their disease. Data regarding prognostic markers in patients in whom hypercalcemia represents the initial clinical manifestation of an underlying malignancy are more limited. In these patients, the histologic type, disease extent and malignancy-specific prognostic features may not yet be known at initial presentation. Prognostic markers obtained from commonly available laboratory parameters may be particularly valuable for early risk assessment while the diagnostic work-up is still in progress.

The prognostic value of m-EASIX in this population may be related to the biological processes reflected by its components. EASIX was originally developed to assess endothelial activation and stress and has subsequently been investigated as a prognostic index in various clinical settings. m-EASIX incorporates CRP, a marker of systemic inflammation, as one of its components [17]. CRP reflects systemic inflammatory activity, whereas LDH is associated with cellular injury, increased cellular turnover and tissue hypoxia [21,22]. Platelets, in turn, participate in the complex interplay among inflammation, coagulation and endothelial function [23]. Thus, rather than reflecting a single laboratory abnormality, m-EASIX may capture the combined effects of systemic inflammation, cellular injury and endothelial-related processes [11,18]. The higher m-EASIX values observed in patients with solid malignancies compared with hematologic malignancies in the present cohort are consistent with the correspondingly higher CRP and neutrophil counts observed in this subgroup (Table 4), suggesting a greater systemic inflammatory burden in solid tumors at presentation.

Although CRP, LDH and platelet count are inherently nonspecific markers, independent studies have directly linked m-EASIX to circulating biomarkers of endothelial activation and coagulopathy, including von Willebrand factor, D-dimer and factor VIII [24], and have shown that m-EASIX correlates with biomarkers of endotheliopathy while outperforming the original EASIX score in predicting severe toxicity [25], supporting its biological plausibility as an indirect marker of endothelial stress. While Log2 m-EASIX showed numerically higher discriminative performance than CRP and NLR, the differences in AUC did not reach statistical significance. Therefore, the present findings do not establish superior discriminatory performance of m-EASIX over these individual markers. Nevertheless, its good overall discrimination and consistent association with 90-day mortality across both malignancy subgroups support its potential utility as a composite prognostic marker in HCM. Larger studies are needed to determine whether m-EASIX provides incremental prognostic value beyond individual inflammatory markers.

This multidimensional nature of m-EASIX may be particularly relevant in the setting of HCM. Although serum calcium is a fundamental parameter for defining the biochemical severity of hypercalcemia, it may not fully reflect the systemic biological burden imposed by the underlying malignancy. Although serum calcium has previously been reported to be associated with short-term prognosis in HCM, corrected serum calcium was not associated with mortality in our cohort, whereas m-EASIX retained its prognostic significance. This finding suggests that prognosis in patients presenting with HCM may not be determined solely by the degree of hypercalcemia. While hypercalcemia represents a prominent metabolic consequence of malignancy, m-EASIX may more broadly reflect the systemic inflammation, cellular injury and host response accompanying the underlying disease. Accordingly, its prognostic value may derive from capturing the overall systemic biological burden of advanced malignancy rather than the severity of hypercalcemia itself.

The prognostic value of EASIX- and m-EASIX-based indices has previously been demonstrated across different malignancies. These indices have been associated with survival outcomes in both hematologic and solid malignancies, including metastatic pancreatic cancer, multiple myeloma, classical Hodgkin lymphoma, urothelial carcinoma and small-cell lung cancer [13,26,27,28,29]. However, previous studies have generally evaluated these indices within specific, predefined malignancy populations. A key contribution of the present study is the evaluation of m-EASIX in a different clinical setting: a heterogeneous population in which HCM was the initial clinical manifestation leading to the diagnosis of an otherwise unrecognized malignancy, rather than a population selected according to a specific cancer type. The association between m-EASIX and mortality in this setting suggests that the index may capture systemic inflammatory, cellular injury and endothelial-related processes relevant across different malignancies.

The differences observed between hematologic and solid malignancies are also noteworthy in this context. Patients with solid malignancies had higher m-EASIX levels and experienced more frequent early mortality. Nevertheless, the association between higher m-EASIX and poorer survival was observed in both hematologic and solid malignancies. Consistent with this finding, no significant interaction was observed between the m-EASIX and malignancy subgroup, providing no evidence that its prognostic association differed between the two groups. The laboratory differences between the groups may reflect their distinct underlying disease biology. The predominance of multiple myeloma within the hematologic malignancy group may explain the higher creatinine and total protein levels observed in these patients, whereas higher CRP levels in solid malignancies may reflect greater systemic inflammatory activity. Despite this biological heterogeneity, the consistent association of higher m-EASIX with poorer survival across both groups supports the possibility that the index captures systemic stress responses shared across different malignancies. Nevertheless, given the limited subgroup sample sizes, these findings should be interpreted cautiously.

A potential clinical advantage of m-EASIX is that it can be readily calculated from routinely measured laboratory parameters. In patients presenting with HCM without a previous cancer diagnosis, risk stratification may be challenging before detailed assessment of tumor type and disease extent has been completed. In this setting, m-EASIX may provide clinically relevant prognostic information and help identify patients at higher risk of mortality while the diagnostic work-up is ongoing. However, the present findings are insufficient to support the use of m-EASIX as a stand-alone clinical decision-making tool. Rather, it may be more appropriately considered a readily available risk indicator that complements clinical assessment.

This study has several limitations. Its retrospective and single-center design may introduce selection and information biases. The limited sample size, particularly within individual malignancy subgroups, restricts the ability to draw malignancy-specific conclusions and limits the generalizability of the subgroup findings. Additionally, both malignancy subgroups were clinically heterogeneous and the formal interaction test may have been underpowered to detect subgroup-specific differences given the limited number of events within each subgroup. The lack of routine PTHrP measurements prevented classification of HCM according to different pathophysiological mechanisms. Similarly, the absence of consistent measurements of 1,25-dihydroxyvitamin D and bone turnover markers limited a more detailed evaluation of the relationship between the underlying mechanisms of hypercalcemia and prognosis. As the primary objective of this study was to evaluate short-term prognosis, overall (long-term) mortality was not analyzed and cause of long-term mortality could not be reliably ascertained retrospectively. Although all-cause mortality was used as the primary endpoint, retrospective classification indicated that 86.1% of deaths within 90 days were disease-related, suggesting that observed mortality predominantly reflected the burden of the underlying malignancy and its complications rather than unrelated causes. In addition, the prognostic performance of m-EASIX and the cutoff used in the survival analyses have not been validated in an independent cohort. These limitations should be taken into account when interpreting the findings and further validation in larger, prospective, multicenter studies is needed.

## 5. Conclusions

In conclusion, m-EASIX may provide clinically relevant prognostic information regarding short-term mortality in patients in whom HCM represents the initial manifestation of an underlying malignancy. Its independent association with mortality, together with the consistent association between higher m-EASIX and poorer survival across both hematologic and solid malignancies, supports its potential value in this heterogeneous patient population. Because it is derived from routinely available laboratory parameters, m-EASIX may offer a practical approach to early risk assessment while the underlying malignancy is being characterized. Further prospective and independent validation is required before its incorporation into routine clinical practice.

## Figures and Tables

**Figure 1:**
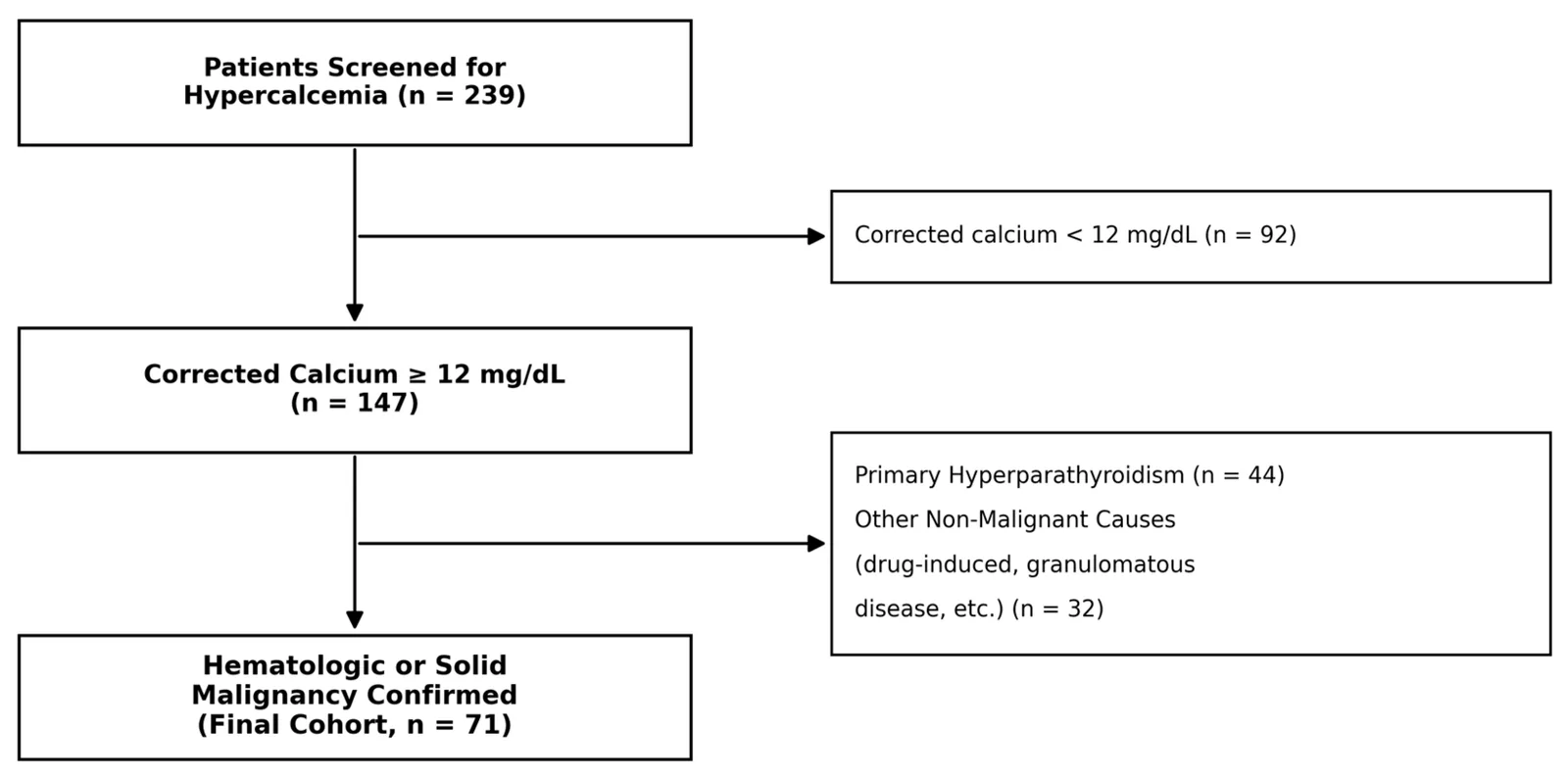
Flowchart of patient selection.

**Figure 2:**
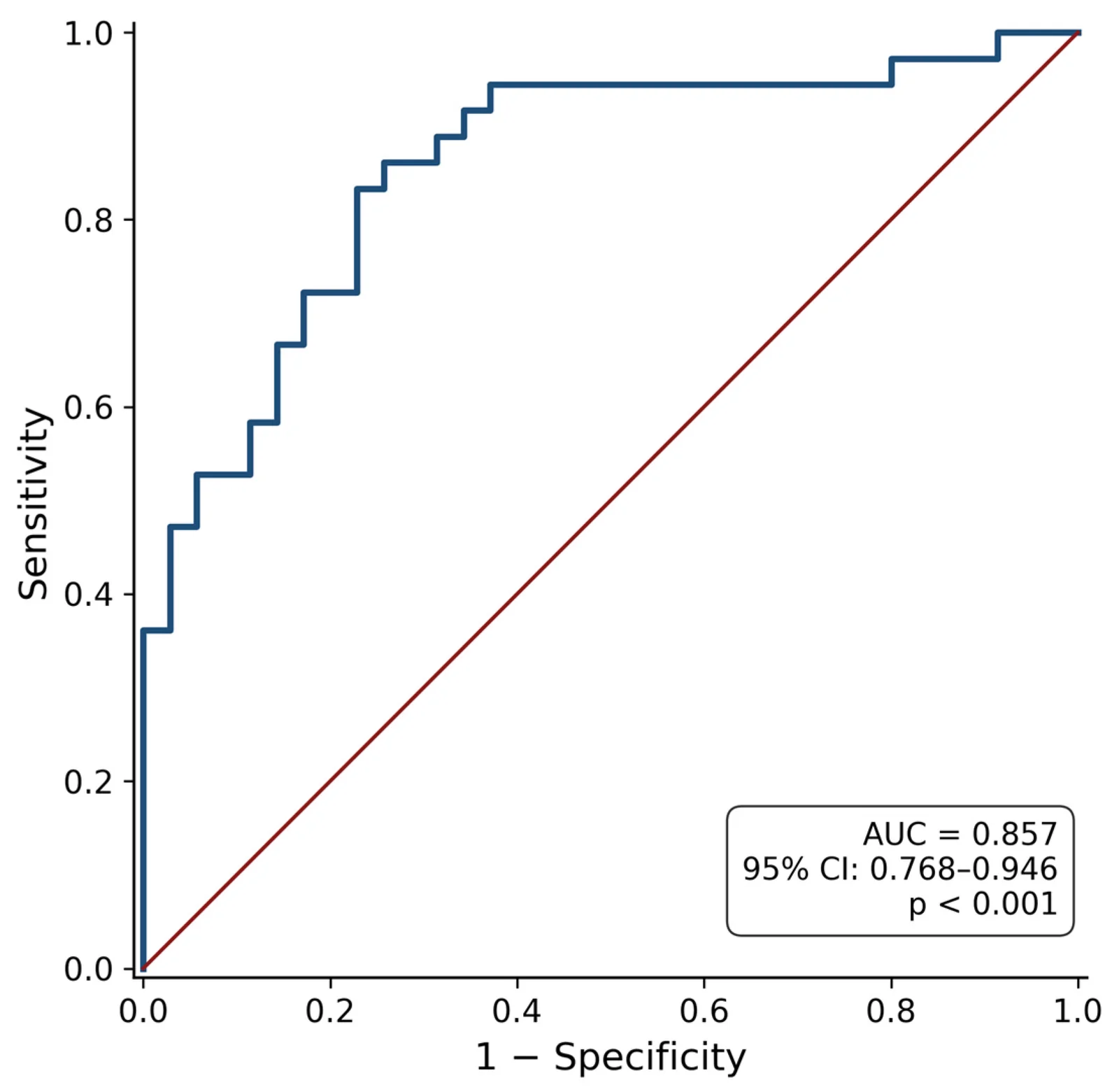
Receiver operating characteristic (ROC) curve for Log2 m-EASIX in predicting 90-day mortality (AUC = 0.857, 95% CI 0.768–0.946, *p* < 0.001).

**Figure 3:**
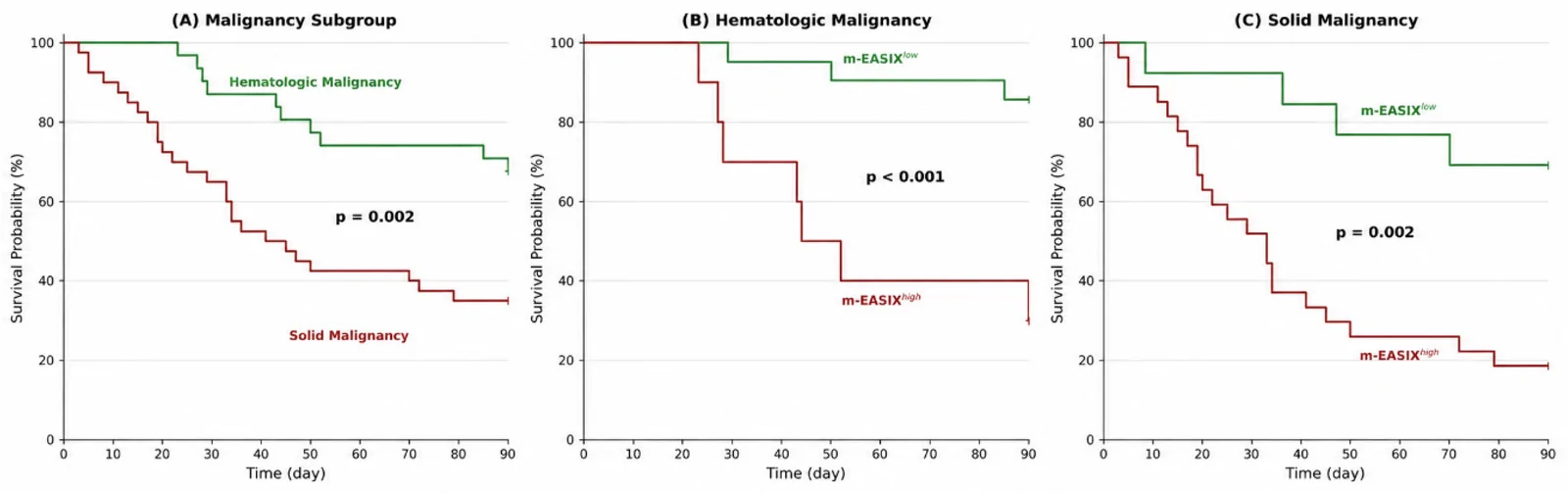
Kaplan–Meier estimates of 90-day survival. (**A**) By malignancy subgroup (hematologic vs. solid). (**B**) By Log2 m-EASIX (low vs. high) within the hematologic malignancy subgroup. (**C**) By Log2 m-EASIX (low vs. high) within the solid malignancy subgroup.

**Table 1:** General patient characteristics (*n* = 71).

Parameters	*n*/Value	
Age, years (mean ± SD)	60.76	± 14.62
Sex, female, *n* (%)	40	(56.3)
Diabetes Mellitus, *n* (%)	17	(23.9)
Hypertension, *n* (%)	34	(47.9)
Hematologic, *n* (%)	31	(43.7)
Solid Malignancy, *n* (%)	40	(56.3)
Hemoglobin, g/dL (mean ± SD)	10.56	±2.23
Neutrophil, 10^3^/µL (median, IQR)	8.11	(4.59–10.41)
Lymphocyte, 10^3^/µL (median, IQR)	1.82	(1.18–2.54)
Monocyte, 10^3^/µL (median, IQR)	0.84	(0.55–1.10)
Platelets, 10^3^/µL (median, IQR)	225	(151.2–305.9)
Ferritin, µg/L (median, IQR)	370.7	(206.8–829.0)
NLR (median, IQR)	4.42	(2.59–6.63)
CRP, mg/L (median, IQR)	64.0	(27.4–134.6)
Creatinine, mg/dL (median, IQR)	1.58	(1.04–2.56)
BUN, mg/dL (median, IQR)	36.4	(26.8–52.0)
Albumin, g/L (mean ± SD)	31.87	±6.06
Total protein, g/L (median, IQR)	66.0	(59.0–75.4)
Corrected calcium, mg/dL (median, IQR)	14.5	(13.3–15.6)
Parathormone, pg/mL (median, IQR)	11.4	(8.05–16.3)
25-OH vitamin D, ng/mL (median, IQR)	14.0	(10.2–17.1)
Phosphorus, mg/dL (median, IQR)	3.2	(2.55–4.0)
Log2 m-EASIX (mean ± SD)	6.19	± 2.48

SD: Standard Deviation, IQR: Interquartile Range, NLR: Neutrophil-to-Lymphocyte ratio, CRP: C-reactive Protein, BUN: Blood Urea Nitrogen, m-EASIX: Modified Endothelial Activation and Stress Index.

**Table 2:** Distribution of diagnosis subgroups (*n* = 71).

Diagnoses	*n*	%
**Hematologic**	**31**	**43.7**
Multiple Myeloma	21	29.6
Lymphoma	8	11.3
Leukemia	2	2.8
**Solid Malignancy**	**40**	**56.3**
Lung	16	22.5
Cancer of Unknown Primary	7	9.9
Stomach	4	5.6
Bladder	3	4.2
Breast	2	2.8
Pancreatic	2	2.8
Ovarian	2	2.8
Intestinal (Colon)	1	1.4
Endometrial	1	1.4
Cholangiocellular	1	1.4
Renal Cell Carcinoma	1	1.4

**Table 3:** Cause of death among patients who died within 90 days (*n* = 36).

Category	*n*	%
**Disease-related**	**31**	**86.1**
Infection/sepsis	19	52.8
Disease progression	11	30.6
Treatment-related toxicity	1	2.8
**Non-disease-related/indeterminate**	**5**	**13.9**
Unrelated cardiac event	1	2.8
Unknown/insufficient documentation	4	11.1

**Table 4:** Comparison of patient characteristics between hematologic and solid malignancies.

Parameters	Hematologic (*n* = 31)	Solid Malignancy (*n* = 40)	*p*-Value
Age, years (mean ± SD)	59.50 ± 13.29	61.74 ± 15.68	0.524 ᵃ
Gender, female, *n* (%)	13 (41.9)	27 (67.5)	0.031 ᵇ
Comorbidity, *n* (%)	22 (71.0)	26 (65.0)	0.594 ᵇ
Hemoglobin, g/dL (mean ± SD)	9.51 ± 2.03	11.38 ± 2.05	<0.001 ᵃ
Neutrophil, 10^3^/µL (median, IQR)	4.65 (3.25–7.83)	9.96 (7.51–13.79)	<0.001 ᶜ
Lymphocyte, 10^3^/µL (median, IQR)	1.71 (1.17–2.54)	1.87 (1.38–2.45)	0.590 ᶜ
Monocyte, 10^3^/µL (median, IQR)	0.84 (0.53–1.29)	0.86 (0.59–1.08)	0.785 ᶜ
Platelets, 10^3^/µL (median, IQR)	223.4 (141.4–281.5)	234.7 (170.8–309.8)	0.327 ᶜ
Ferritin, µg/L (median, IQR)	279.0 (162.7–645.0)	382.5 (282.8–880.0)	0.122 ᶜ
NLR (median, IQR)	2.82 (1.86–4.50)	6.11 (3.95–8.40)	<0.001 ^c^
CRP, mg/L (median, IQR)	24.1 (9.95–81.1)	90.25 (48.4–139.7)	0.002 ᶜ
Creatinine, mg/dL (median, IQR)	2.11 (1.46–3.10)	1.23 (0.93–1.83)	<0.001 ᶜ
Corrected calcium, mg/dL (median, IQR)	13.90 (12.75–15.45)	14.90 (13.60–15.55)	0.147 ᶜ
25-OH Vitamin D, ng/mL (median, IQR)	15.60 (10.70–17.10)	12.85 (9.52–17.48)	0.281 ᶜ
Parathormone, pg/mL (median, IQR)	12.30 (8.20–16.75)	10.85 (8.07–16.07)	0.562 ᶜ
Albumin, g/L (mean ± SD)	31.55 ± 6.86	32.12 ± 5.44	0.694 ᵃ
Total protein, g/L (median, IQR)	79.0 (60.0–103.2)	63.0 (56.5–69.3)	0.001 ᶜ
Log2 m-EASIX (mean ± SD)	5.08 ± 2.56	7.05 ± 2.05	<0.001 ᵃ
30-day mortality, *n* (%)	3 (9.7)	16 (40.0)	0.004 ᵇ
90-day mortality, *n* (%)	10 (32.3)	26 (65.0)	0.013 ᵇ

^a^: Independent Samples *t*-test, ^b^: Chi-square Test, ^c^: Mann–Whitney U Test, NLR: Neutrophil-to-Lymphocyte ratio, CRP: C-Reactive Protein, SD: Standard Deviation, IQR: Interquartile Range, m-EASIX: Modified Endothelial Activation and Stress Index.

**Table 5:** Univariate and multivariate logistic regression analysis for 90-day mortality.

Factor	Univariate Analysis				Multivariate Analysis			
	OR	Lower	Upper	*p*	OR	Lower	Upper	*p*
Age, years	1.045	1.008	1.084	0.018				
Gender (Male vs. Female)	1.484	0.578	3.809	0.412				
Comorbidity	2.625	0.935	7.369	0.067				
Hemoglobin, g/dL	1.007	0.816	1.242	0.948				
Neutrophil, 10^3^/µL	1.234	1.074	1.419	0.003				
Lymphocyte, 10^3^/µL	0.800	0.492	1.301	0.369				
Monocyte, 10^3^/µL	1.942	0.748	5.047	0.173				
Platelets, 10^3^/µL	0.999	0.996	1.003	0.696				
Ferritin, µg/L	1.001	1.000	1.002	0.078				
NLR	1.315	1.089	1.589	0.005				
CRP, mg/L	1.018	1.008	1.027	<0.001				
Creatinine, mg/dL	0.889	0.674	1.173	0.406				
BUN, mg/dL	1.018	0.996	1.041	0.117				
Serum corrected calcium, mg/dL	1.055	0.803	1.384	0.702				
Parathormone, pg/mL	0.968	0.935	1.003	0.071				
25-OH Vitamin D, ng/mL	1.038	0.983	1.097	0.183				
Phosphorus, mg/dL	0.896	0.634	1.265	0.531				
Total Protein, g/L	0.971	0.946	0.997	0.031				
Wbc, 10^3^/µL	1.172	1.050	1.309	0.005	1.133	0.985	1.303	0.080
Hematologic vs. Solid Malignancy	3.900	1.443	10.544	0.007				
LDH, U/L	1.002	1.000	1.004	0.056				
Albumin, g/L	0.885	0.806	0.971	0.010	0.887	0.780	1.009	0.068
**Log2 m-EASIX**	**2.153**	**1.487**	**3.117**	**<0.001**	**1.962**	**1.368**	**2.815**	**<0.001**

OR: Odds Ratio, Wbc: White Blood Cell Count, LDH: Lactate Dehydrogenase, CRP: C-Reactive Protein, BUN: Blood Urea Nitrogen, m-EASIX: Modified Endothelial Activation and Stress Index.

## Data Availability

The datasets analyzed during this study are available from the corresponding author upon reasonable request.

## Supplementary Materials

The following supporting information can be downloaded at: https://www.mdpi.com/article/10.3390/jcm15197382/s1, Table S1. Bootstrap stability of variable selection. Table S2. Standard versus Firth penalized logistic regression. Table S3. Subgroup-specific univariate analysis of 90-day mortality. Table S4. Comparison of the discriminative performance of Log2 m-EASIX, CRP and NLR for 90-day mortality.
