# Supplementary material for: Prognostic Value of the Modified Endothelial Activation and Stress Index (m-EASIX) for 90-Day Mortality in Patients with Moderate-to-Severe Hypercalcemia of Malignancy
Source: J Clin Med. 2026 Sep 23;15(19):7382. doi: 10.3390/jcm15197382 (PMC13665553; doi:10.3390/jcm15197382)
Supplement: Supplementary file 1 [file jcm-15-07382-s001.zip › jcm-4534819-supplementary.pdf]

Supplementary Table S1. Bootstrap stability of variable selection.

Variable	*Bootstrap Selection Frequency (%)*
Log2 m-EASIX	***98.1***
Albumin	*51.7*
White blood cell count	*38.6*
Age	*28.8*
Neutrophil-to-lymphocyte ratio	*27.9*
Total protein	*20.4*
Malignancy subgroup (hematologic vs. solid)	*16.7*

Bootstrap resampling (1000 iterations) was performed to assess the stability of variable selection in the multivariable backward elimination procedure. In each resample, seven candidate variables (age, malignancy subgroup, total protein, albumin, white blood cell count, neutrophil-to-lymphocyte ratio and Log2 m-EASIX) were entered and the backward likelihood-ratio elimination procedure was repeated. The proportion of resamples in which each variable was retained in the final model is reported as its selection frequency. Log2 m-EASIX was retained in 98.1% of resamples, substantially more frequently than any other candidate variable.

Supplementary Table S2. Standard versus Firth penalized logistic regression.

Variable	Method	OR	95% CI	p
**Log2 m-EASIX**	**Standard MLE**	**1.962**	**1.368–2.815**	**<0.001**
**Log2 m-EASIX**	**Firth Penalized**	**1.823**	**1.309–2.540**	**<0.001**
White blood cell count	Standard MLE	1.133	0.985–1.303	0.080
White blood cell count	Firth Penalized	1.115	0.972–1.279	0.120
Albumin	Standard MLE	0.887	0.780–1.009	0.068
Albumin	Firth Penalized	0.899	0.796–1.014	0.083

To assess whether small-sample bias may have inflated the effect estimates of the final multivariable model, the model (white blood cell count, albumin and Log2 m-EASIX) was refitted using Firth's penalized (bias-reduced) logistic regression, which corrects for the small-sample bias inherent in standard maximum likelihood estimation. Effect estimates from the Firth-penalized model were similar to those from standard maximum likelihood estimation, and Log2 m-EASIX remained independently and significantly associated with 90-day mortality (p<0.001).

Supplementary Table S3. Subgroup-specific univariate analysis of 90-day mortality.

Variable	Hematologic Malignancy, p	Solid Malignancy, p
**Log2 m-EASIX**	**0.012**	**0.004**
C-reactive protein	0.012	0.064
Neutrophil-to-lymphocyte ratio	0.049	0.171
Albumin	0.101	0.018
White blood cell count	0.054	0.130
Age	0.119	0.092
Total protein	0.565	0.079

Univariate logistic regression analyses for 90-day mortality were performed separately within the hematologic malignancy (n=31) and solid malignancy (n=40) subgroups for each candidate variable. Log2 m-EASIX was the only variable that reached statistical significance (p<0.05) in both subgroups. Given the limited subgroup sample sizes, these findings should be interpreted as exploratory and hypothesis-generating.

Supplementary Table S4. Comparison of the discriminative performance of Log2 m-EASIX, CRP and NLR for 90-day mortality.

Marker	AUC	95% CI	DeLong z*	p
**Log2 m-EASIX**	**0.857**	**0.768–0.946**	**Reference**	**—**
**CRP**	**0.794**	**0.684–0.904**	**1.544**	**0.123**
**NLR**	**0.752**	**0.638–0.867**	**1.482**	**0.138**

*Pairwise comparison with Log2 m-EASIX using DeLong's test, m-EASIX: Modified Endothelial Activation and Stress Index, CRP: C-Reactive Protein, NLR: Neutrophil-to-Lymphocyte ratio, AUC: Area under the Curve, CI: Confidence Interval
